# Harsh environmental conditions promote cooperative behavior in an epiphytic fern

**DOI:** 10.1080/15592324.2024.2335453

**Published:** 2024-03-30

**Authors:** Kahurangi Cronin, Ian Hutton, K.C. Burns

**Affiliations:** a School of Biological Sciences, Victoria University of Wellington, Wellington, New Zealand; b Lord Howe Island Museum, Lord Howe Island, Australia

**Keywords:** Sociality, colonial living, cooperation, Platycerium, behavior

## Abstract

Harsh, unpredictable environments are known to favor cooperative groups in animals. Whether plants exhibit similar relationships is unknown. Staghorn ferns (*Platycerium bifurcatum*, Polypodiaceae) are epiphytes that form cooperative groups which build communal water and nutrient ‘nests’ at the tops of trees, a habitat characterized by water and nutrient stress. We conducted field observations to test whether staghorn ferns continue to live in large, reproductively active groups after they become dislodged from the canopy and fall to the forest floor, where they are less limited by water and nutrient deprivation. To rule out the potentially confounding effects of light limitation on the forest floor, we also conducted a multi-year glasshouse experiment where we transplanted individual plants into soil and onto vertically oriented boards under standardized light conditions. Results from field observations showed that dislodged colonies formed smaller groups that reproduced less than epiphytic colonies. Results from the glasshouse experiment showed that even when growing in full sun, terrestrial individuals tended to remain solitary, while epiphytic individuals tended to recruit new individuals into colonies. Results also showed that plants growing in potting soil and exposed to full sunlight sporulated more heavily than plants growing epiphytically. However, localities that are characterized by both elevated soil and light resources are generally not available to staghorn ferns in the wild, perhaps with the exception of large, epiphytic colonies with well-developed nests at the top of tree canopies. Overall results indicate that the harsh environmental conditions at the tops of trees trigger the formation of colonies in staghorn ferns, similarly to group living animals.

Recent research has linked the evolution of cooperative living to harsh, unpredictable environmental conditions^1–4^ but see.^5^ Group living can be adaptive in a variety of different ways in harsh environments, including facilitating thermoregulation,^6,7^ providing greater vigilance to predators,^8,9^ or increasing hunting efficiency.^10,11^ Although cooperation-environment relationships are an emerging paradigm in animals,^12,13^ whether plants exhibit similar relationships is unknown.

The staghorn fern (*Platycerium bifurcatum*, Polypodiaceae) is an epiphytic fern with a distinctive life history (Figure 1). Although it is a common ornamental around the world, it is native to the east coast of Australia, New Guinea, and parts of Southeast Asia. It occurs primarily in the forest canopy, attached to the trunks or branches of trees. However, it can also be found on the forest floor after plants become dislodged from the canopy above.

**Figure 1.**
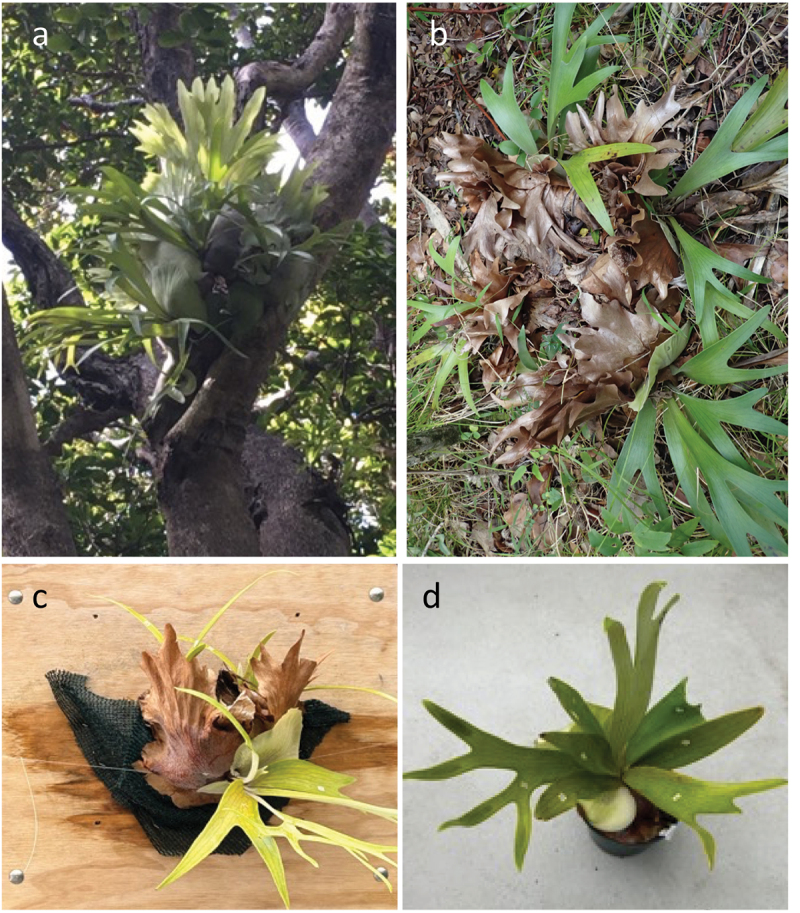
(a) Staghorn fern colony (*Platycerium bifurcatum*) growing epiphytically on Lord Howe Island. (b) Staghorn fern colony growing terrestrially on Lord Howe Island. (c) Experimental (glasshouse) colony growing epiphytically. (d) Experimental (glasshouse) colony growing terrestrially.

Tree branches are difficult places for plants to live. Without access to water and nutrients in soil on the forest floor, epiphytes are regularly water and nutrient stressed see.^14^ Correspondingly, epiphytes have evolved a variety of strategies to mediate water and nutrient deprivation. For example, orchids have evolved specialized stems (pseudobulbs) and root tissues (velamen radicum) that store water, as well as mutualistic associations with fungi that fix atmospheric nitrogen (mycorrhiza).

*Platycerium bifurcatum* appears to have solved the problem differently. When growing epiphytically, it forms cooperative groups of individual plants that work together to build a ‘nest’, which serves as a communal water and nutrient store^15^. Although colonies are comprised of clones, they are occasionally comprised of unrelated individuals.^16^ Individual plants recruit continuously into colonies and produce two types of fronds. ‘Strap’ fronds are longer, narrower, and can be both reproductively and photosynthetically active. On the other hand, ‘nest’ fronds are larger, more elliptical in shape and die at maturity. However, following mortality, they continue to trap and store rainwater, airborne detritus and leaf litter within nests.

The morphology of staghorn ferns varies not only between frond types but also among individual plants within colonies, resulting in strong vertical gradients in frond size and shape. Plants at the top of colonies produce strap fronds that are often reproductively active, while those at the bottom of colonies often fail to reproduce entirely, leading to strong reproductive skew. Strap fronds at the top of colonies are also lobed, stiff, vertically orientated, and curved like rain gutters to capture rainwater and then channel it toward the center of colonies (de Bock et al. *unpubl)*. Conversely, strap fronds at the bottom are less numerous, smaller, flaccid and hang pendulously. Plants at the top of colonies also produce larger, fan-shaped nest fronds that articulate with other colony members to trap water and airborne detritus, leading to the development of a water and nutrient ‘nest’. Nest fronds at the very top of colonies also appear to have a defensive function. Each folds over the nest opening as they decay, helping to protect colonies from invasion by other plant species (Cronin et al. *unpubl*.). Conversely, nest fronds at the bottom of colonies are smaller, spongy in texture, and store water that is channelled to them from colony members above,^15^ (Cronin et al. *unpubl*).

Variability in the morphology of fronds, both within and among individual plants, promotes the division of labor within colonies to combat water and nutrient stress. These attributes (overlapping generations, reproductive division of labor and cooperative care of young) are similar to those of eusocial animals. However, the extent to which the life history attributes of staghorn ferns parallel those of eusocial animals has yet to be elucidated.

Here, we conducted field observations to test whether staghorn ferns form densely populated cooperative groups that are more reproductively active when growing epiphytically, relative to when they become dislodged and come to reside in soil on the forest floor. To account for differences in light conditions between habitats, we also transplanted individual plants onto vertically oriented boards and into potting soil to test whether plants growing terrestrially form smaller social groups than those growing epiphytically when growing under standardized (full sun) light conditions.

## Methods

Field observations were conducted on Lord Howe Island (LHI), an eroded shield volcano located approximately 550 km from the east coast of Australia in the Tasman Sea (31°33‘31“S, 159°05’09“E). LHI was chosen for study because lowland areas across much of the island are dominated by stunted tropical dry forest that supports large populations of staghorn ferns. On the east coast of Australia, staghorn ferns typically grow in tall rainforest trees and are therefore difficult to access. However, on LHI they frequently grow within arm’s reach due to the stunted nature of low elevation forest.

We located all staghorn fern colonies growing both epiphytically and terrestrially within 5 m of either side of a series of narrow foot tracks that collectively spanned approximately 3.5 km in total length. ‘Epiphytic’ colonies always grew on the trunks or branches of trees and their root systems were never connected to soil on the forest floor. All colonies classified as ‘terrestrial’ were found residing on the forest floor. All terrestrial colonies showed clear evidence of originating in the canopy above and subsequently became dislodged. Although staghorn ferns sometimes grow on rocks on the forest floor (i.e. lithophytically), we restricted our attention to colonies that were located away from bare rock surfaces. Dislodged colonies usually had a hollow shape, that surrounded dead, decaying branches, and were articulated with downed material from the host.

To test for differences in colony density between habitats, colony volume was measured by multiplying their vertical height, maximum horizontal length, and horizontal width perpendicular to the length measurement (cm^3^), and colony size was measured by counting the total number of individual plants present within each colony. A general linear model was then conducted using colony size (the total number of individuals) as the dependent variable, habitat as a fixed factor with two levels (epiphytic vs terrestrial), and colony volume as a covariate. Colony size was logarithm transformed (+1) to conform to assumptions. This and all subsequent analyses were conducted in the R environment.^17^

To test whether epiphytic colonies were more reproductively active than terrestrial colonies, the total number of reproductively active fronds (i.e. fronds with sori) produced by all individual plants within each colony was quantified. A general linear model was then conducted using the total number of reproductively active fronds within colonies as the dependent variable, habitat as a fixed factor, and colony size as a covariate.

While harsh soil conditions are a hallmark of epiphytic habitats,^14^ water and nutrient stress is much reduced in the soil on the forest floor below. However, light attenuates from the forest canopy to the ground below, creating strong vertical gradients in photosynthetically active radiation.^18^ Therefore, epiphytic colonies are undoubtedly exposed to greater light availability than terrestrial colonies growing below. To control for the potential effects of differences in light availability between habitats on colony density and reproductive output, we conducted a glasshouse experiment where plants were grown both epiphytically (on vertically orientated boards) and terrestrially (in potting soil) under standardized (full sun) light environments (Figure 1).

The experiment was conducted in glasshouse facilities at Victoria University of Wellington, New Zealand. Although *P. bifurcatum* is not native to New Zealand,^19^ it is commonly grown ornamentally above 38°S and can be purchased from private growers. In August 2021, 20 individual plants were sourced from large, epiphytic colonies growing in the Auckland region, transported to Wellington, and allocated individually to one of two habitats. Ten plants were randomly allocated to the ‘terrestrial’ habitat and 10 plants were randomly allocated to the ‘epiphytic’ habitat. Plants assigned to terrestrial habitat were placed into individual pots (12 cm tall and 12 cm in diameter) filled with commercially available KiwiGardener potting mix™. Plants assigned to the ‘epiphytic’ habitat were attached to vertically oriented boards (40 cm × 40 cm) onto a small, circular ball of commercially available *Sphagnum* moss approximately 10 cm in diameter using plastic mesh and monofilament line. Plants were watered to saturation every 2–3 days and exposed to full sunlight. Temperatures ranged between 18–23°C and natural temperature extremes beyond this range were mediated by automatic temperature control systems. Plants that died in the process of transplantation were immediately replaced.

Two and a half years later, in December 2023, the number of new recruits and the number of reproductively active fronds were quantified on each replicate. The total number of new recruits was defined as the total number of newly appearing individual plants (i.e. with a separate rhizome) from each original plant. The number of reproductively active fronds was defined as the total number of strap fronds with sori, which are always produced on the tips of each frond. Separate Poisson linear models were then conducted to test for differences in the number of recruits and reproductively active fronds between habitats.

## Results

**Figure 2.**
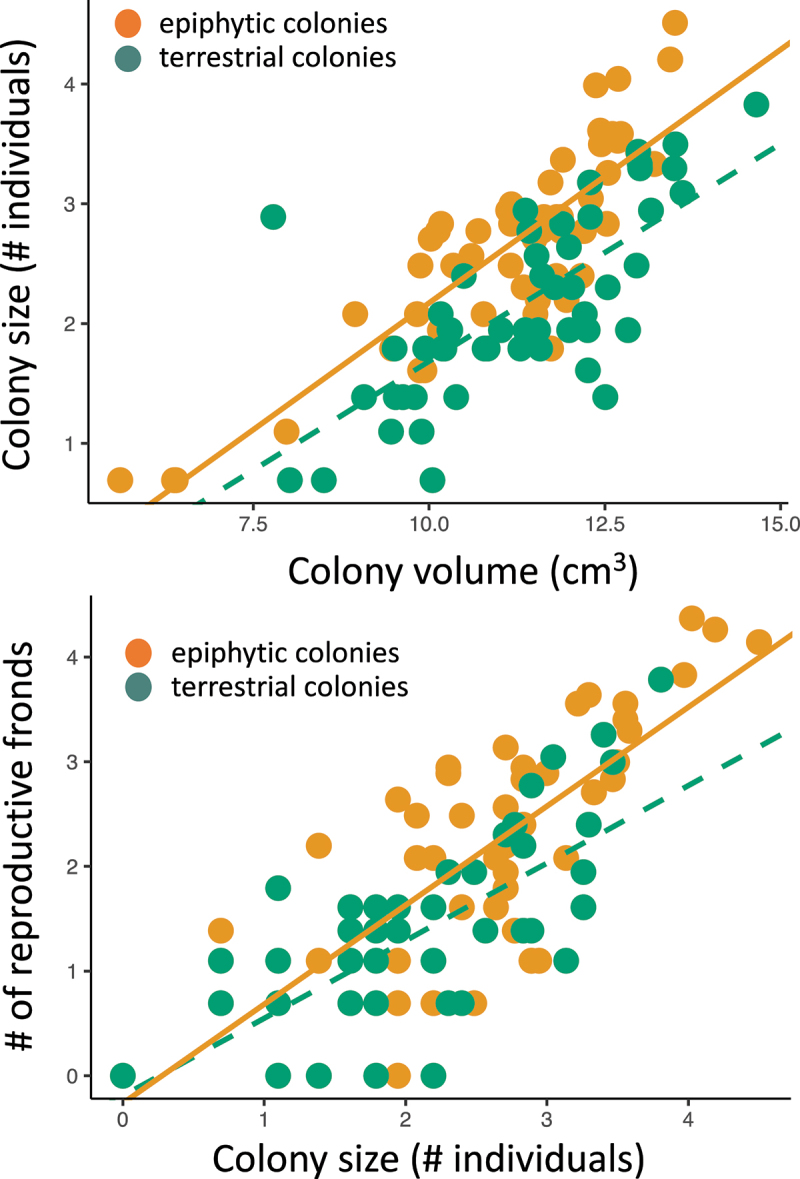
(Top) differences in the density of individuals within colonies growing epiphytically (orange points) and after becoming dislodged on the forest floor (green points). (Bottom) differences in the rate of spore production between colonies growing epiphytically (orange points) and after becoming dislodged on the forest floor (green points).

One hundred and five colonies were encountered (*n* = 52 terrestrial and 53 epiphytic) during field sampling. Epiphytic colonies had higher population densities and generally reproduced more than colonies that had become dislodged naturally and fell to the forest floor (Figure 2). The first general linear model showed that the number of individuals present in colonies increased with colony volume (*t* = 13.196, *p* < .001) and was higher in the epiphytic habitat (*t* = −5.852, *p* < .001). The slope of relationships for each habitat was statistically similar (*t* = −0.991, *p* = .324). The second linear model showed that spore production increased with number of individuals present in colonies (*t* = 12.277, *p* < .001) and was higher in the epiphytic habitat (*t* = −2.581, *p* = .011). The slopes for each habitat were again statistically similar (*t* = −1.309, *p* = .193).

**Figure 3.**
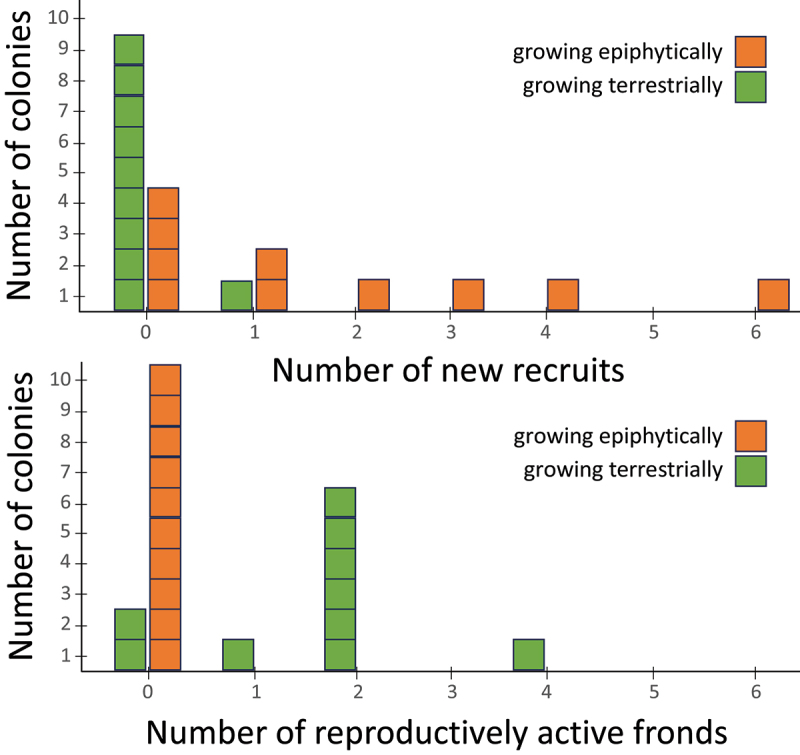
Rates of colony development (top graph) and spore production (bottom graph) in 10 staghorn fern individuals planted epiphytically (on vertically oriented boards, orange symbols) and 10 individuals planted terrestrially (in soil, green symbols). When exposed to standardized (full sun) conditions terrestrial colonies tend to remain solitary, while those growing epiphytically, without access to elevated water and nutrients, begin to form colonies. Plants exposed to high light, water, and nutrient conditions (conditions which are generally not available innature) begin to reproduce via spores.

Results from the glasshouse experiment showed that terrestrial colonies continued to grow solitarily, yet reproduced more intensely, when exposed to full sunlight (Figure 3). The Poisson generalized linear models showed that epiphytic individuals tended to recruit greater numbers of new colony members asexually than terrestrial individuals (*p* = .006), and that terrestrial individuals sporulated more heavily (*p* = .003).

## Discussion

Group living has been linked to adverse environmental conditions in many different types of animals see.^3^ For example, cooperatively breeding birds are more prevalent in regions that experience harsh, unpredictable environments.^1,2,4^ Results from this study indicate that similar relationships may also occur in plants.

Relationships between environmental conditions and group living could arise via several mechanisms. They could arise evolutionarily via natural selection, or they also can arise ecologically when group living animals settle in regions with harsh environments.^2^ Previous work has typically focused on circumstances where solitary species begin to form collaborative groups when exposed to adverse environmental conditions. However, results from this study illustrate the opposite pattern – that group living is selected against in more benign soil conditions.

Epiphytic colonies growing in the field reproduced more heavily (i.e. they produced more sporulating fronds) than dislodged colonies that fell from the canopy above and came to reside on the forest floor. This suggests that staghorn ferns are more fit when growing within cooperative colonies under the harsh water and nutrient conditions at the tops of trees. Yet a seemingly opposite pattern was observed in the glasshouse. When growing under standardized light conditions in the glasshouse (full sun), terrestrially based plants reproduced more heavily than epiphytic plants. Reproductive fitness therefore appears to be highest in plants exposed to both elevated light and soil availability. However, habitats characterized by unlimited light, water and nutrient availability generally do not exist stably in forested environments,^18^ with one possible exception – large staghorn fern colonies with fully developed water and nutrient ‘nests’ growing in full sun at the tops of trees.

Overall results showed that staghorn ferns form collaborative groups to mediate the effects of water and nutrient stress at the tops of trees. When grown in more benign soil conditions they tend to grow solitarily. Therefore, relationships between harsh environmental conditions and the formation of collaborative social groups may be more widespread than previously appreciated and may span both plant and animal kingdoms.

## Supplementary Material

ESM.xlsx
